# Atheroprotective Pulsatile Flow Induces Ubiquitin-Proteasome–Mediated Degradation of Programmed Cell Death 4 in Endothelial Cells

**DOI:** 10.1371/journal.pone.0091564

**Published:** 2014-03-13

**Authors:** Cheng Ge, Jiantao Song, Liang Chen, Lin Wang, Yifei Chen, Xinxin Liu, Yu Zhang, Lining Zhang, Mei Zhang

**Affiliations:** 1 Key Laboratory of Cardiovascular Remodeling and Function Research, Chinese Ministry of Education and Chinese Ministry of Health, Department of Cardiology, Shandong University Qilu Hospital, Jinan, Shandong, People’s Republic of China; 2 Department of Emergency, Shandong University Qilu Hospital, Jinan, Shandong, People’s Republic of China; 3 Department of Immunology, School of Medicine, Shandong University, Jinan, Shandong, People’s Republic of China; Georgia Regents University, United States of America

## Abstract

**Objectives:**

We recently found low level of tumor suppressor programmed cell death 4 (PDCD4) associated with reduced atherosclerotic plaque area (unpublished). We investigated whether atheroprotective unidirectional pulsatile shear stress affects the expression of PDCD4 in endothelial cells.

**Methods and Results:**

*En face* co-immunostaining of the mouse aortic arch revealed a low level of PDCD4 in endothelial cells undergoing pulsatile shear stress. Application of unidirectional pulsatile shear stress to human umbilical vein endothelial cells (HUVECs) decreased PDCD4 protein but not mRNA level. Immunoprecipitation revealed that pulsatile shear stress induced the coupling of ubiquitin with PDCD4 expression. The phosphatidyl inositol 3-kinase (PI3K)/Akt pathway was involved in this ubiquitin-proteasome–mediated degradation of PDCD4. Gain of function and loss of function experiments showed that PDCD4 induced turnover (proliferation and apoptosis) of HUVECs. Low PDCD4 level was associated with reduced proliferation but not apoptosis or phosphorylation of endothelial nitric oxide synthase caused by pulsatile shear stress to help maintain the homeostasis of endothelial cells.

**Conclusions:**

Pulsatile shear stress induces ubiquitin-proteasome–mediated degradation of PDCD4 via a PI3K/Akt pathway in HUVECs. PDCD4 induces turnover (proliferation and apoptosis) of HUVECs. Low PDCD4 level is associated with reduced proliferation for maintenance of HUVEC homeostasis under pulsatile shear stress.

## Introduction

Programmed cell death 4 (PDCD4) is an important tumor suppressor in the development of various human cancers [1] and inhibits translation rather than transcription. Specifically, the PDCD4 protein combines directly with the mRNA coding region of the target gene (*MYB/c-MYB*) to block translation [2]. It can also compete with eukaryotic translation initiation factor (eIF)4G and RNA for eIF4A binding and trap eIF4A in an inactive conformation to inhibit translation initiation via its two highly conserved MA3 domains [3]–[6]. Thus, PDCD4 regulates molecules functioning during tumor cell proliferation, apoptosis, transformation, invasion and autophagy [1], [7], [8]. Although PDCD4 in general suppresses the development and progression of tumors, its specific biological functions differ by cell type [8].

PDCD4 also plays a role in cardiovascular cell biology by inhibiting proliferation and inducing apoptosis of most cardiovascular cells, including vascular smooth muscle cells [9], [10], cardiac myocytes [11] and fibroblasts [12], and repressing contractile gene expression in vascular smooth muscle cells [13]. However, the action of PDCD4 in endothelial cells is unclear.

Atherosclerosis plaques preferentially develop at arterial branches and curvatures under low and oscillatory or even static shear stress induced by disturbed blood flow as opposed to in straight parts, featuring protective unidirectional pulsatile shear stress [14], [15]. Vascular endothelial cells, as a monolayer in direct contact with the flowing blood, bear the most of the wall shear stresses and have important homeostatic functions in response to stress [16]. Ample evidence shows that pro-atherosclerotic disturbed flow induces sustained activation of atherogenic genes in endothelial cells to promote their oxidation, inflammation, cell cycle progression and proliferation, whereas pulsatile shear stress tends to maintain endothelial cells in a quiescent and less proliferative state with a low level of oxidation and inflammation [16], [17].

Our recent study of PDCD4 and ApoE double-deficient mice (PDCD4−/−ApoE−/−) fed a high-cholesterol diet found that knockout of PDCD4 was associated with reduced atherosclerosis plaque areas (unpublished data), so PDCD4 downregulation might protect arteries against atherosclerosis. To determine whether PDCD4 plays a role in the response of vascular endothelial cells to atheroprotective pulsatile shear stress, we examined PDCD4 expression in endothelial cells under shear stress both *in vivo* and *in vitro* and investigated the involved mechanisms.

## Materials and Methods

### Materials

Rabbit monoclonal antibody (mAb) for PDCD4; rabbit polyclonal antibody (pAb) for ubiquitin; rabbit mAb for phospho-eNOS (Ser1177); rabbit pAb for eNOS, cleaved caspase-3, and caspase-3; mouse mAb for phospho-Akt (Ser473) and phosphatase and tensin homologue deleted on chromosome ten (PTEN); rabbit mAb for Akt, β-transducin repeat-containing protein (β-TrCP) and p21^Waf1/Cip1^; mouse mAb for β-actin; MG-132; and LY294002 were from Cell Signaling Technology (Danvers, MA, USA). Rabbit pAbs for phospho-p70-S6K (T412) and p70-S6K were from Immunoway (Newark, NJ, USA). Rat mAb for PECAM-1 and protein A/G plus agarose were from Santa Cruz Biotechnology (Santa Cruz, CA, USA). Rabbit pAb for PDCD4 (phospho S67) were from Abcam (Cambridge, UK). Mouse mAb for GAPDH and FITC-conjugated goat anti-rat IgG secondary antibodies were from ZSGB-BIO (Beijing). Negative control microRNA inhibitor, miR-21 inhibitor, negative control microRNA mimics and miR-21 mimics were from GenePharma (Shanghai). Alexa Fluor 647-labeled goat anti-rabbit and goat anti-mouse IgG secondary antibodies were from Beyotime Institute of Biotechnology (Haimen, Jiangsu, China). Rat tail collagen I was from BD Biosciences (San Jose, CA, USA). Lactacystin was from Sigma (St. Louis, MO, USA). All other chemicals of reagent grade were from Invitrogen (Life Technologies, Carlsbad, CA, USA), unless otherwise noted.

### *En Face* Staining of Mice Aortas

Five C57BL/6 mice (male, 8–10 weeks old, 20–25 g) were purchased from Peking University (Beijing) and kept on a 12-hr light/12-hr dark cycle, with food and water freely available. All animal experiments were performed in accordance with the Animal Management Rules of the Chinese Ministry of Health (document No 55, 2001) and with the approval of the Animal Care Committee of Shandong University.

C57BL/6 mice were deeply anesthetized with 0.8% (wt/vol) pentobarbital sodium and transcardially perfused with 30 mL lukewarm saline, followed by 100 mL of 4% paraformaldehyde. After perfusion, aortas were harvested and postfixed in this fixative solution for 4 hr and then subjected to *en face* immunostaining. Briefly, tissues were washed with phosphate buffered saline (PBS) and the adventitia was removed carefully. Aortas were longitudinally dissected with use of microdissecting scissors, immediately blocked with 5% (vol/vol) bovine serum albumin for 0.5 hr, then incubated with specific primary antibodies rat anti-CD31 mAb (1∶50) and rabbit anti-PDCD4 mAb (1∶600) at 4°C overnight, then FITC-conjugated anti-rat IgG (1∶50) and Alexa Fluor 647-conjugated anti-rabbit IgG (1∶200) secondary antibodies. Samples were counterstained with 4′,6-diamidino-2-phenylindole (DAPI) (Beyotime Institute of Biotechnology) for nuclei, rinsed 3 times in PBS, mounted with glycerol:PBS (1∶1) and photographed under a laser-scanning confocal microscope with a 40X objective (oil immersion) (Model LSM710, Zeiss, Jena, Germany).

### Cell Culture

Human umbilical vein endothelial cells (HUVECs) were isolated from fresh human umbilical cords by trypsin perfusion. The cell pellet was resuspended in a culture medium consisting of medium 199 (M199; Hyclone, Thermo Fisher Scientific Inc., Waltham, MA, USA) supplemented with 20% (vol/vol) fetal bovine serum (FBS) (Hyclone), 2 ng/ml fibroblast growth factor-2, and 1% (vol/vol) penicillin/streptomycin. HUVECs (from passage 4 to 7) were cultured in M199 containing 10% FBS with 5% CO_2_ at 37°C for 3 days and then seeded onto glass slides (75 by 25 mm; Flexcell International Corp., Hillsborough, NC, USA) precoated with collagen I. Secondary cultures were used in experiments within 2 days after reaching confluence (1–2×10^5^ cells/cm^2^).

The study protocol conformed to the ethical guidelines of the 1975 Declaration of Helsinki with the approval of the Institutional Medical Ethics Committee of Qilu Hospital, Shandong University. All donors provided written informed consent.

### Flow Apparatus

Cells plated on collagen-coated slides were exposed to unidirectional pulsatile shear stress (1 Hz) of 12 dynes/cm^2^ or oscillatory shear stress (1 Hz) of 0±4 dynes/cm^2^ in a Streamer (Flexcell International Corp.), the parallel plate flow chamber, which was incorporated into a closed loop containing culture medium and kept in an incubator with 5% CO_2_ at 37°C. A MasterFlex peristaltic pump (Cole Parmer, Vernon Hills, IL, USA) was applied to run the medium in the loop, and the type of shear stress was determined by the computer-controlled osci-flow apparatus (Flexcell International Corp.). Cells cultured under static conditions were controls. In some experiments, HUVECs were incubated with a specific inhibitor for 1 hr before and during exposure to flow.

### Western Blot Analysis

Cells were lysed with Cell lysis buffer for Western and IP (Beyotime Institute of Biotechnology) and 1 mM phenylmethanesulfonyl fluoride. The total cell lysates (30 µg of protein) were separated by SDS-PAGE (10% separating, 4% stacking) and after incubation overnight at 4°C with the designated antibodies (1∶1000), protein levels were analyzed by use of the LAS-4000 luminescent image analyzer (Fujifilm, Stamford, CT, USA). Band densities were analyzed by use of Adobe Photoshop CS3 (Adobe Systems, San Jose, CA, USA).

### Immunofluorescence

Cells were fixed with 4% paraformaldehyde and permeabilized with 0.1% Triton X-100 for 15 min. After incubation overnight at 4°C with anti-PDCD4 antibodies (1∶600), cells were washed with PBS and incubated for 1 hr with Alexa 647-conjugated (1∶200) secondary antibodies and washed with PBS. Nuclei were stained with DAPI for 8 min. Cells were examined under a laser-scanning confocal microscope with a 40X objective (oil immersion) (Model LSM710, Zeiss, Jena, Germany).

### RNA Isolation and Real-time PCR

Total RNA was extracted by use of TRIzol reagent. RNA concentrations were determined by use of the NanoDrop ND-1000 spectrophotometer (Nanodrop Technologies, Wilmington, DE, USA). The first-strand cDNA was synthesized from 2 µg total RNA with use of random primers and the PrimeScript RT reagent kit (Takara Bio Inc.; Otsu, Shiga, Japan). Real-time PCR involved the SYRB Premix Ex Taq kit (Takara Bio Inc.). Primers for PDCD4 were forward, 5′-TGAGCACGGAGATACGAACGA-3′ and reverse, 5′-GCTAAGGACACTGCCAACACG-3′; and β-actin forward, 5′-CGTGCGTGACATTAAGGAGA-3′ and reverse, 5′-CACCTTCACCGTTCCAGTTT-3′. β-actin was used as housekeeping gene. The relative mRNA expression level was assessed by the 2^−ΔΔCt^ method.

### Immunoprecipitation

Cells were lysed with Cell lysis buffer for Western and IP (Beyotime Institute of Biotechnology) and 1 mM phenylmethanesulfonyl fluoride. The same amount of protein from each sample was incubated with cognate antibodies for 2 hr at 4°C, then with protein A/G plus agarose overnight at 4°C with gentle rotation. The agarose-bound immunoprecipitates were rinsed and collected by centrifugation and incubated with the SDS-PAGE sample loading buffer (2X) (Beyotime Institute of Biotechnology), and subjected to SDS-PAGE and western blot analysis.

### Transient Transfection

*pEGFP-C1-Mock* or *pEGFP-C1-PDCD4* plasmids were constructed and kindly provided by Dr. Olubunmi Afonja (New York University, New York). Transfection of vectors involved the Lipofectamine 2000 method (Invitrogen).

The sequence for the miR-21 inhibitor was 5′-UCAACAUCAGUCUGAUAAGCUA-3′ and those for miR-21 mimics were 5′-UAGCUUAUCAGACUGAUGUUGA-3′ and 5′-AACAUCAGUCUGAUAAGCUAUU-3′. The sequence for PDCD4 small interfering RNA (siRNA) was 5′-GUGUUGGCAGUAUCCUUAG-3′ and those for β-TrCP siRNA were 5′-GCACUUGCGUUUCAAUAAUTT-3′ and 5′-AUUAUUGAAACGCAAGUGCTT-3′. MicroRNA inhibitor, mimics and siRNA interference involved the Lipofectamine 2000 method (Invitrogen).

### Bromodeoxyuridine (BrdU) Incorporation Assay

Proliferation was detected using the 5-Bromo-2′-deoxy-uridine Labeling and Detection Kit I (Roche Diagnostics Corp, Indianapolis, IN, USA). Cells were treated with BrdU (10 µM) for 6 hr before harvesting and were rinsed and fixed for 30 min at −20°C with Ethanol fixative. After washed in the Washing buffer, they were stained with Anti-BrdU working solution at 4°C overnight, washed and incubated with Alexa Fluor 647-conjugated anti-mouse antibody for 30 min at 37°C and DAPI for 8 min at room temperature as a counterstain for the nucleus. The stained cells were examined using a laser-scanning confocal microscope with a 20X objective (Model LSM710, Zeiss, Jena, Germany). Proliferation was assessed based on the percentage of nuclei exhibiting BrdU incorporation.

### Apoptosis Detection (Terminal Deoxynucleotidyl Transferase-mediated UTP Nick End Labeling, TUNEL)

Apoptosis was detected using the ApopTag Plus Peroxidase *In Situ* Apoptosis Detection Kit (Millipore, Billerica, MA, USA). Cells were washed with PBS (pH 7.4) and then fixed in 1% paraformaldehyde for 10 min at room temperature and permeabilized in precooled ethanol:acetic acid 2∶1 for 5 min at −20°C. They were washed and immersed in the Equilibration Buffer for 10 sec and incubated with the Working Strength TdT Enzyme at 37°C for 1 hr. After incubation with the Stop/Wash Buffer for 10 min at room temperature, ECs were incubated with the Anti-Digoxygenin Peroxidase Conjugate and Peroxidase Substrate to detect signs of apoptosis, staining brown. Counterstaining was carried out with 0.5% (w:v) methyl green. Apoptosis was assessed based on the percentage of TUNEL positive nuclei.

### Statistical Analysis

Data are expressed as mean±SEM. Statistical analysis involved independent Student *t* test for comparing 2 groups and one-way ANOVA for multiple comparisons. *P*<0.05 was considered statistically significant.

## Results

### PDCD4 Expression is Maintained at a Low Level in Areas of Atheroprotective Flow *in vivo*

**Figure 1:**
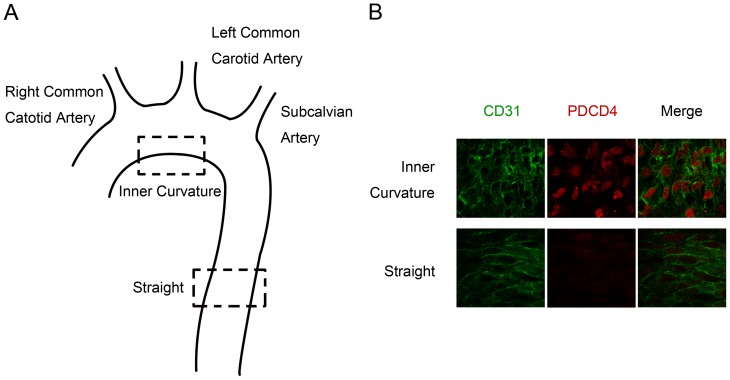
Downregulation of programmed cell death 4 (PDCD4) in the pulsatile flow area in native circulation *in vivo* in mice. (A) Inner curvature and straight segment of the aorta. (B) Laser-scanning confocal microscopy of *en face* co-immunostaining for CD31, for endothelial cells, and PDCD4 in the inner curvature of the aortic arch and straight segment of thoracic aorta of C57BL/6 mice (n = 5).

To investigate whether atheroprotective and atheroprone shear stress have different effects on PDCD4 expression in vascular endothelial cells *in vivo*, we examined the aortic arch and straight segment of thoracic aortas (Figure 1A) from C57BL/6 mice by *en face* co-immunostaining for CD31 (for endothelial cells) and PDCD4. Endothelial cells were observed elongated and aligned with the longitudinal axis of the vessel in the straight segments of thoracic aortas, where shear stress is high and pulsatile; they showed a polygonal morphology in the inner curvature of aortic arches, where disturbed flow occurs with relatively low and oscillating shear stress or even under static conditions, in accordance with previous findings [16]. Level of PDCD4 was high in endothelial cell nuclei in the inner curvature of aortic arches, but was low in the straight segments of thoracic aortas (Figure 1B). Thus, pulsatile shear stress in the native circulation maintains PDCD4 expression at a low level in endothelial cells *in vivo*.

### Pulsatile Shear Stress Downregulates PDCD4 Protein Level in HUVECs

**Figure 2:**
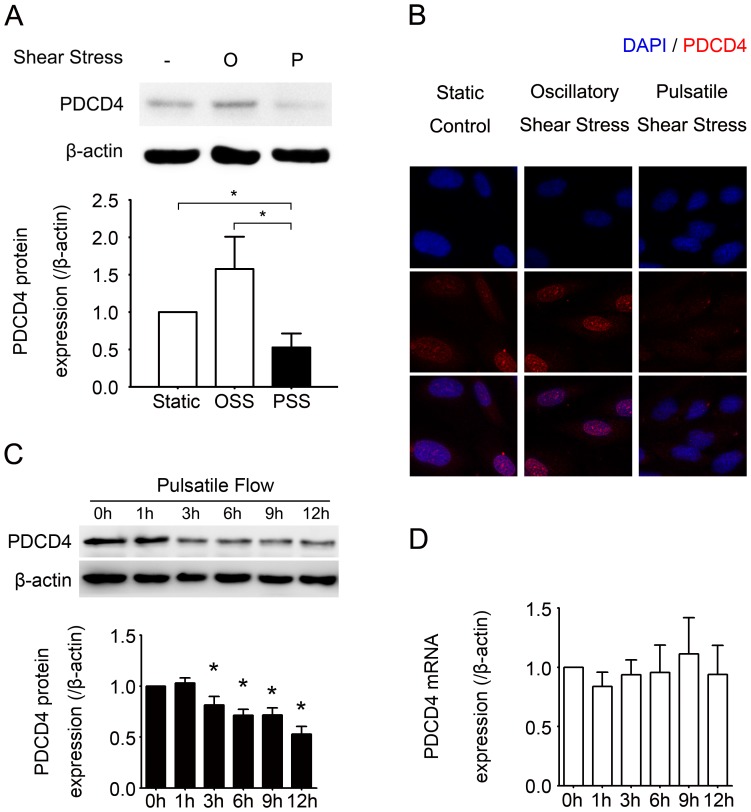
Post-transcriptional downregulation of PDCD4 expression with pulsatile shear stress in human umbilical vein endothelial cells (HUVECs). (A) Western blot analysis of protein level of PDCD4 in HUVECs kept under static conditions or subjected to oscillatory shear stress (OSS) at 0±4 dynes/cm^2^ or unidirectional pulsatile shear stress (PSS) at 12 dynes/cm^2^ for 12 hr. (B) Confocal laser-scanning microscopy of PDCD4 protein by immunofluorescence. DAPI was used to stain nuclei. Results are representative of 3 independent experiments. (C, D) Western blot analysis of protein level and real-time quantitative PCR analysis of mRNA level, respectively, of PDCD4 in HUVECs kept under static conditions or subjected to pulsatile shear stress at 12 dynes/cm^2^ for various times. Quantitative data are mean±SEM from 3 independent experiments and are presented as change in band density from control cells normalized to internal protein levels. *P<0.05 vs. 0 h in (C).

To investigate whether different shear stresses play a role in modulating PDCD4 expression in endothelial cells, we applied unidirectional pulsatile shear stress at 12 dynes/cm^2^ or oscillatory shear stress at 0±4 dynes/cm^2^ over 12 hr to HUVECs; cells cultured under static conditions were controls. The protein level of PDCD4 was reduced by nearly half with pulsatile shear stress as compared with static control cells (*P* = 0.002) and even more as compared with cells under oscillatory shear stress (*P* = 0.001) (Figure 2A). Immunofluorescence of HUVECs revealed decreased PDCD4 level in nuclei of cells under pulsatile shear stress as compared with oscillatory shear stress or static conditions (Figure 2B). PDCD4 protein level decreased gradually over time with pulsatile shear stress (Figure 2C), with only a slight and non-significant increase in mRNA level (Figure 2D). Therefore, pulsatile shear stress decreases PDCD4 expression post-transcriptionally in HUVECs.

### Pulsatile Shear Stress Reduces PDCD4 Protein Level via the Ubiquitin-proteasome Pathway

**Figure 3:**
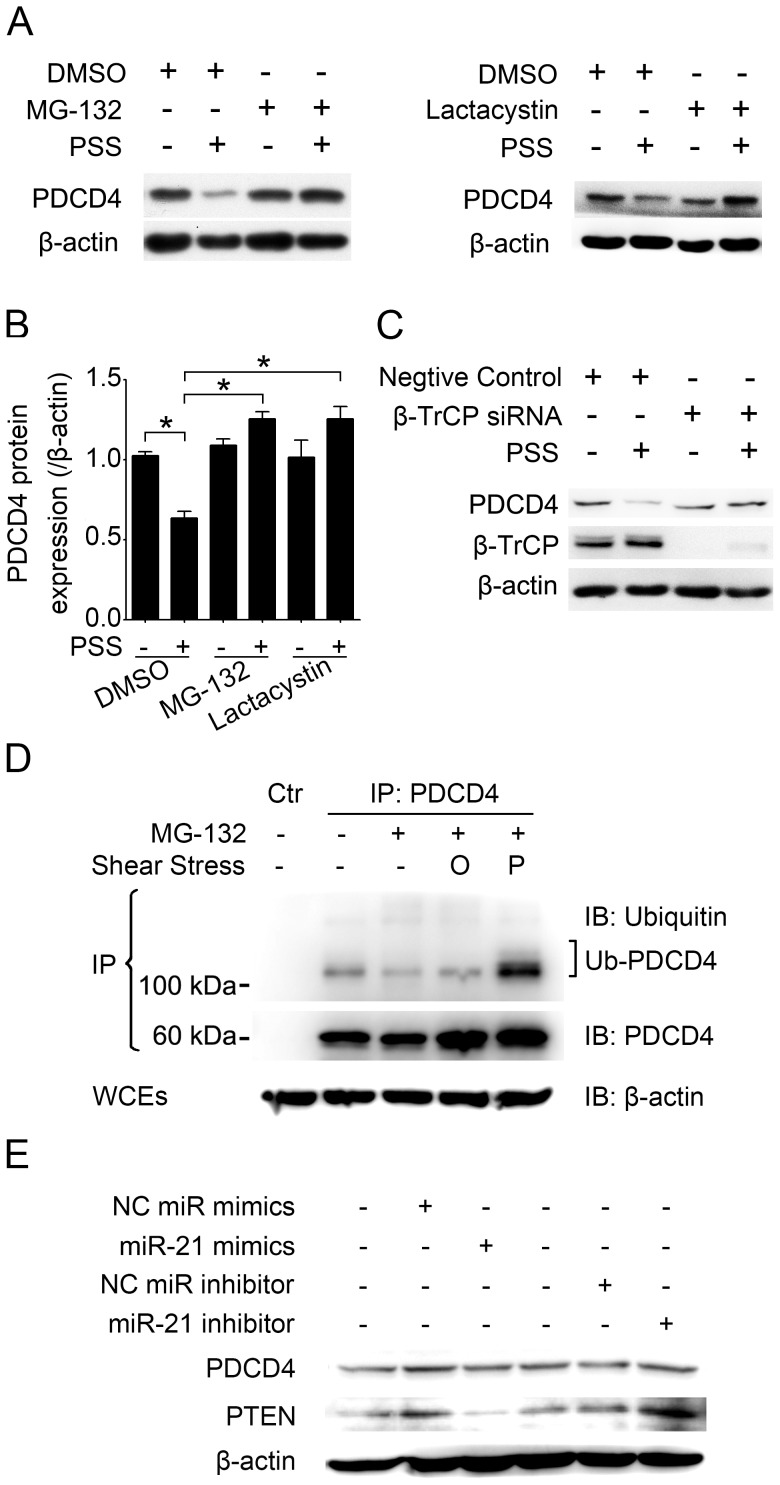
Ubiquitin-proteasome–mediated degradation of PDCD4 by pulsatile shear stress. (A–C) Western blot analysis of PCDC4 and β-transducin repeat-containing protein (β-TrCP) levels in HUVECs kept under static conditions or subjected to pulsatile shear stress (PSS) at 12 dynes/cm^2^ for 12 hr. Cells were pretreated with DMSO, MG-132 (10 µM) or lactacystin (10 µM) for 1 hr before and during the shearing process. For β-TrCP downregulation, cells were transfected with β-TrCP small interfering RNA (siRNA) or negative control. Quantitative data are mean±SEM from 3 independent experiments and are presented as change in band density from control cells normalized to internal protein levels. *P<0.05. (D) HUVECs were kept under static conditions or subjected to oscillatory shear stress (O) at 0±4 dynes/cm^2^ or pulsatile shear stress (P) at 12 dynes/cm^2^ for 12 hr and treated with DMSO or MG-132 during the last 4 hr before harvesting; whole-cell extracts (WCEs) were immunoprecipitated (IP) with the PDCD4-specific primary antibody and protein A/G plus agarose, followed by immunoblotting (IB) with antibodies for the indicated proteins. Ub-PDCD4 stands for ubiquitinated PDCD4. Ctr stands for negative control. (E) Western blot analysis of protein levels of PDCD4 and phosphatase and tensin homologue deleted on chromosome ten (PTEN) in HUVECs transfected with miR-21 mimics, miR-21 inhibitor; negative control (NC) microRNA (miR) mimics or inhibitor. Results in (C) and (E) are representative of 3 independent experiments.

The ubiquitin-proteasome pathway mediates the degradation of PDCD4 under various conditions [18], [19], and miR-21 negatively regulates PDCD4 expression translationally in various tumor cells [20] and other cardiovascular cells [9], [11], [12]. We investigated whether these pathways were involved in decreased PDCD4 level with pulsatile shear stress. The proteasome inhibitor MG-132 (10 µM) or lactacystin (10 µM) was added into the medium 1 hr before and during the shearing process with HUVECs, with dimethyl sulfoxide (DMSO) as a control. We also transfected the cells with β-TrCP siRNA. β-TrCP is an ubiquitin ligase involved in degradation of PDCD4 [18], [19]. Either proteasome inhibitors or downregulation of β-TrCP completely rescued the PDCD4 level downregulated with pulsatile shear stress, but neither affected PDCD4 level under static conditions (Figure 3A–C). To confirm this result, we performed coimmunoprecipitation assays with MG-132 applied during the last 4 hr before cells were harvested to block the degradation function of proteasomes and retain the ubiquitinated proteins. Pulsatile shear stress significantly induced ubiquitination of PDCD4 as compared with oscillatory shear stress or static conditions (Figure 3D). Hence, pulsatile shear stress reduces PDCD4 protein level via the ubiquitin-proteasome pathway. We also transfected HUVECs with miR-21 mimics or miR-21 inhibitor (PTEN as a positive control, which is confirmed to be a target of miR-21 in HUVECs [21]), with no alteration of PDCD4 expression (Figure 3E), so the ubiquitin-proteasome pathway is more implied in the mechanism.

### Phosphatidyl Inositol 3-kinase (PI3K)/Akt Pathway Mediates the Degradation of PDCD4 by Pulsatile Shear Stress

**Figure 4:**
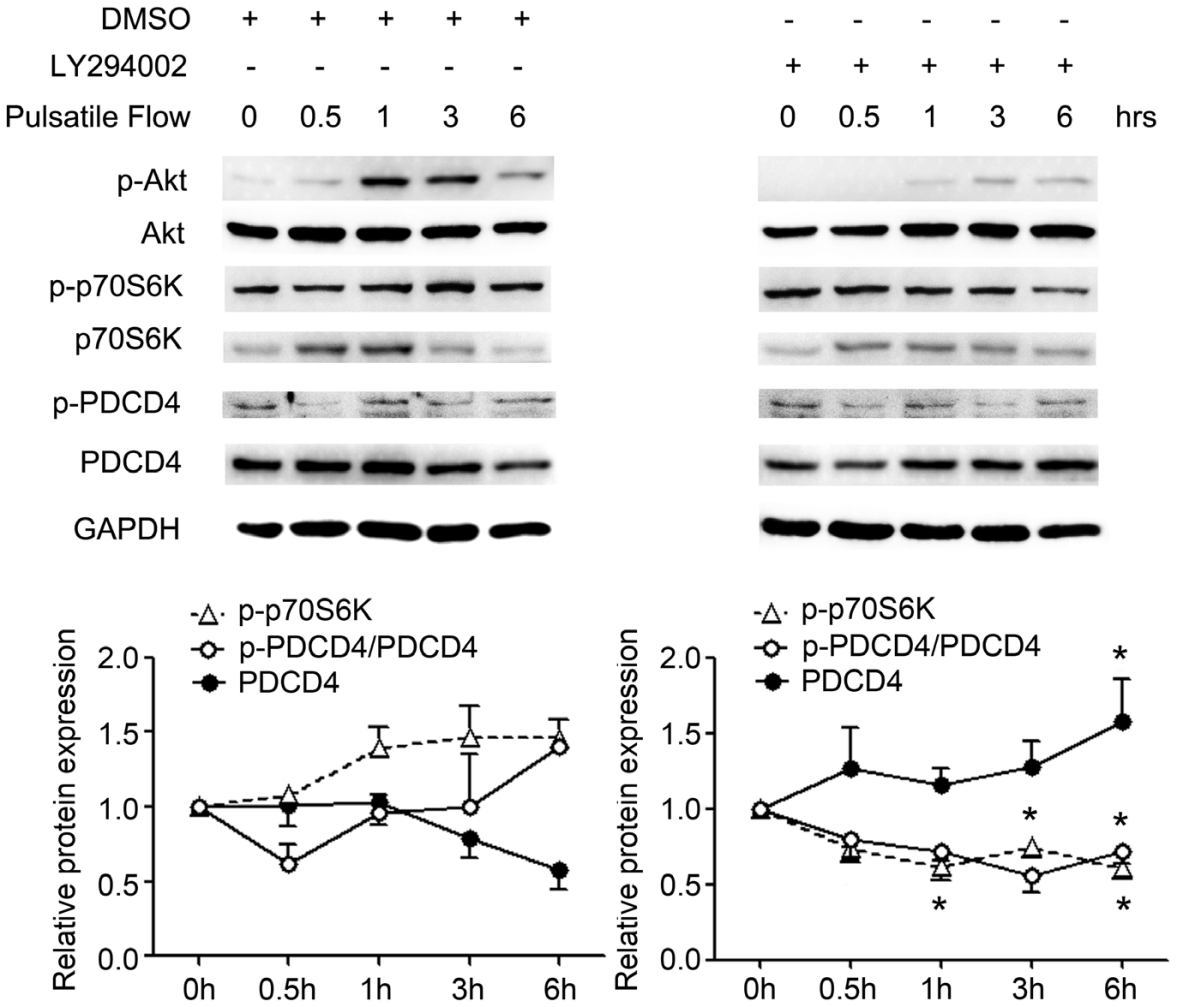
PI3K/Akt pathway is involved in degradation of PDCD4 with pulsatile shear stress. Western blot analysis of protein levels in the PI3K/Akt pathway and PDCD4 in HUVECs pretreated with DMSO or PI3K inhibitor LY294002 (10 µM) for 1 hr and subjected to pulsatile shear stress at 12 dynes/cm^2^ for the indicated times. Quantitative data are mean±SEM from 3 independent experiments and are presented as change in band density from control cells normalized to internal protein levels. *P<0.05 vs. DMSO-treated cells sheared for a same period.

Pulsatile shear stress activates several signaling pathways including the PI3K/Akt pathway [22]; in tumor cells, phosphorylated Akt can activate the 70-kDa ribosomal protein S6 kinase (p70-S6K), which may phosphorylate PDCD4 for ubiquitin binding [18], [19]. To further investigate whether the PI3K/Akt pathway couples PDCD4 degradation with pulsatile shear stress, HUVECs were pretreated with the specific PI3K inhibitor LY294002 (10 µM) or vehicle control for 1 hr, then were subjected to pulsatile shear stress for various times. Along with inhibition of phosphorylation of Akt, activation of p70-S6K and phosphorylation of PDCD4 were blocked, whereas PDCD4 level was retained or even upregulated (Figure 4), which indicates that the PI3K/Akt pathway mediates the degradation of PDCD4 by pulsatile shear stress.

### PDCD4 Induces Turnover (Proliferation and Apoptosis) of HUVECs

**Figure 5:**
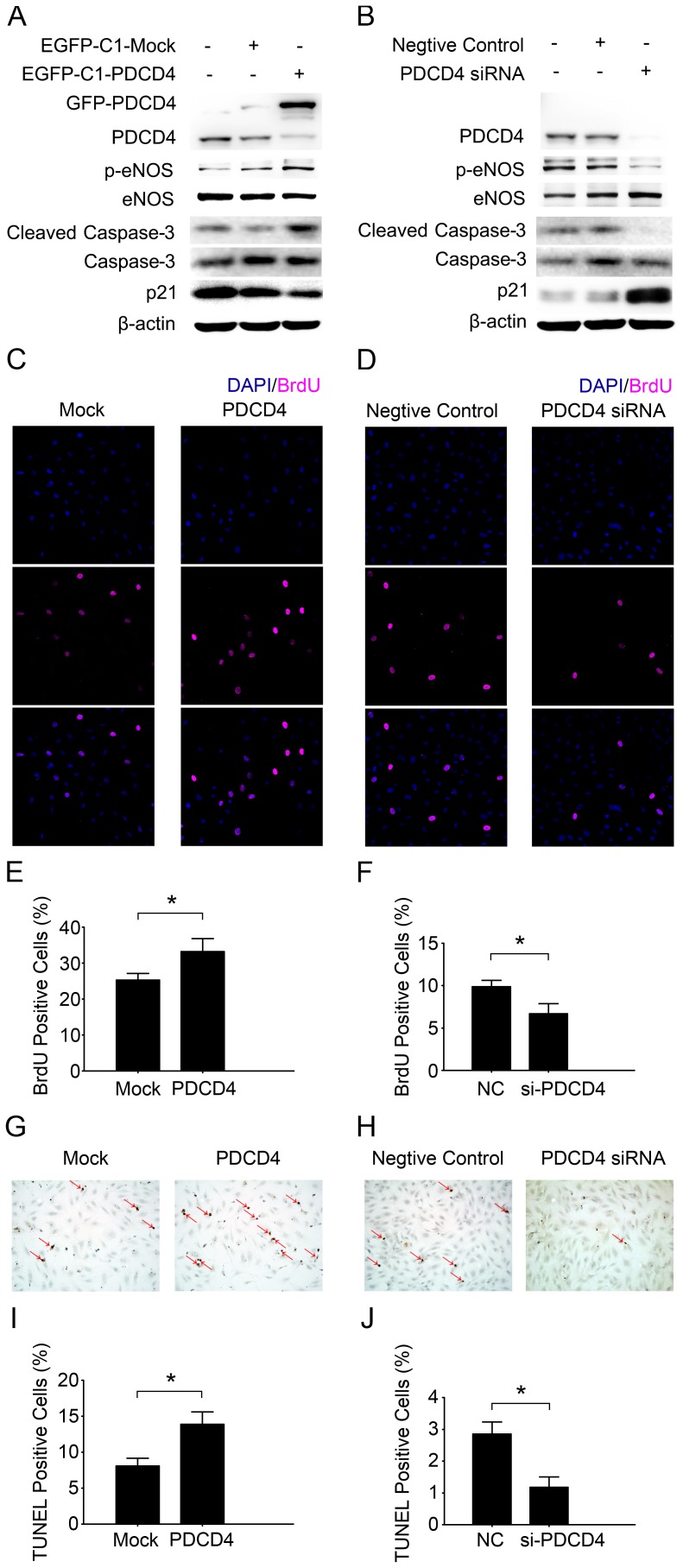
PDCD4 induces turnover (proliferation and apoptosis) of HUVECs. HUVECs were transfected with *pEGFP-C1-Mock* (Mock), *pEGFP-C1-PDCD4* (PDCD4), negative control (NC) or PDCD4 siRNA (si-PDCD4). (A, B) Western blot analysis of protein levels as indicated. Results are representative of 3 independent experiments. (C, D) Confocal laser-scanning microscopy of BrdU by immunofluorescence (magenta). BrdU was added into the medium 6 hr before harvesting. DAPI was used to stain nuclei (blue). (E, F) The percentage of BrdU positive cells was calculated. (G, H) HUVECs with stained nuclei (brown) were considered TUNEL positive (red arrows). (I, J) The percentage of TUNEL positive cells was calculated. Quantitative data in (E, F, I, J) are mean±SEM from 3 independent experiments. *P<0.05.

We next investigated the role of PDCD4 in maintaining HUVEC homeostasis. We examined the expression of p21^Waf1/Cip1^, a cyclin-dependent kinase inhibitor that suppresses cell proliferation [23]; cleaved or active caspase-3, the final executioner of most apoptosis [24]; and phosphorylated endothelial nitric oxide (NO) synthase (eNOS), the key enzyme for NO production in endothelial cells that maintains vascular functions [25]. The occurrence of proliferation was detected by BrdU incorporation assay while apoptosis by TUNEL technique. Overexpression of PDCD4 by transfecting cells with *pEGFP-C1-PDCD4* downregulated p21^Waf1/Cip1^ protein level as compared with mock treatment but upregulated that of cleaved caspase-3, increased proliferation, apoptosis and phosphorylated eNOS (Figure 5A, C, E, G, I). Downregulation of PDCD4 by siRNA did the opposite (Figure 5B, D, F, H, J). Therefore, PDCD4 induces turnover of HUVECs.

### Downregulation of PDCD4 by Pulsatile Shear Stress Rescues p21^Waf1/Cip1^ and Reduces Proliferation to Slow the Turnover of HUVECs

**Figure 6:**
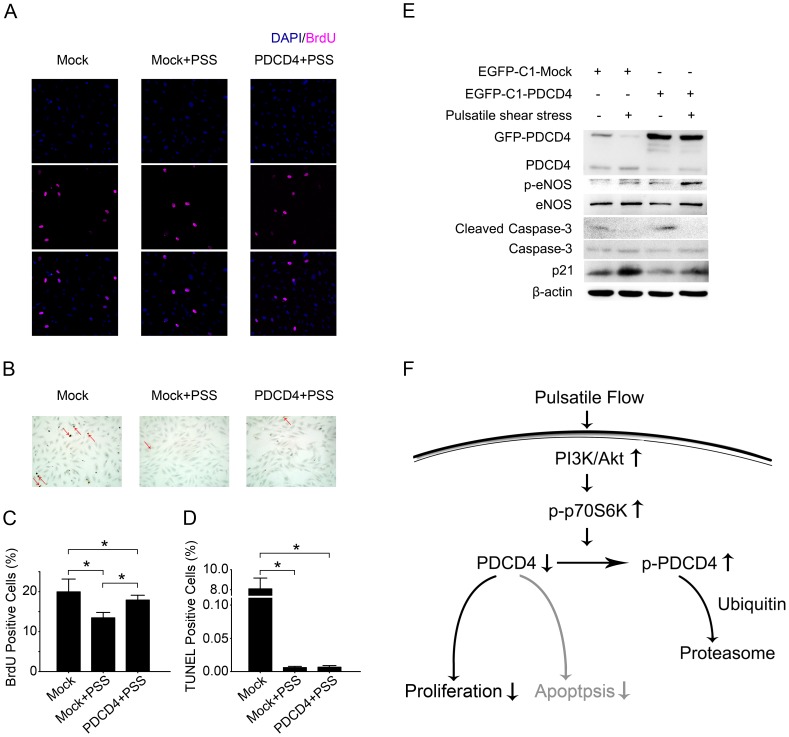
Downregulation of PDCD4 by pulsatile shear stress rescues p21^Waf1/Cip1^ and reduces proliferation to slow the turnover of HUVECs. HUVECs transfected with *pEGFP-C1-Mock* (Mock) or *pEGFP-C1-PDCD4* (PDCD4) were subjected to pulsatile shear stress (PSS) or kept under static conditions. (A) Confocal laser-scanning microscopy of BrdU by immunofluorescence (magenta). BrdU was added into the medium 6 hr before harvesting. DAPI was used to stain nuclei (blue). (B) HUVECs with stained nuclei (brown) were considered TUNEL positive (red arrows). (C) The percentage of BrdU positive cells was calculated. (D) The percentage of TUNEL positive cells was calculated. Quantitative data in (C, D) are mean±SEM from 3 independent experiments. *P<0.05. (E) Western blot analysis of indicated protein levels. Results are representative of 3 independent experiments. (F) The black arrows show that pulsatile shear stress reduces proliferation in part via ubiquitin-proteasome–mediated degradation of PDCD4 through a PI3K/Akt/p70-S6K pathway. The gray arrows show that PDCD4 alone induces apoptosis of HUVECs under static conditions.

HUVECs transfected with *pEGFP-C1-PDCD4* or *pEGFP-C1-Mock* were subjected to pulsatile shear stress or kept under static conditions. Pulsatile shear stress induced p21^Waf1/Cip1^ protein expression and phosphorylated eNOS, suppressed activation of caspase-3, reduced proliferation and apoptosis (Figure 6A, B, C, D, E). Overexpression of PDCD4 blocked the induction of p21^Waf1/Cip1^ and partly rescued proliferation reduction with pulsatile shear stress but not the suppression of cleaved caspase-3 or reduction of apoptosis (Figure 6A, B, C, D, E). Overexpression of PDCD4 with pulsatile shear stress doubled the phosphorylation of eNOS (Figure 6E).

## Discussion

We recently found that downregulation of the tumor suppressor PDCD4 was associated with reduced atherosclerotic plaque area (unpublished data). To investigate whether atheroprotective pulsatile shear stress affects the expression of PDCD4 in endothelial cells, we performed *in vivo* and *in vitro* experiments and found that atheroprotective pulsatile shear stress maintained PDCD4 protein at a low level. In endothelial cells, the PDCD4 level was kept low via upregulation of ubiquitin-proteasome–mediated degradation of PDCD4 through the PI3K/Akt pathway and induction of p21^Waf1/Cip1^ to help maintain the homeostasis of endothelial cells.

Accumulating evidence suggests that PDCD4 plays an important role in cardiovascular diseases. PDCD4 was found induced by H_2_O_2_ in cardiac myocytes [11], which suggests a role of PDCD4 in heart diseases such as myocardial infarction. An *in vivo* experiment in zebrafish showed that protection of PDCD4 against miR-21 binding plays a necessary role in cardiac valvulogenesis [26]. Our previous study revealed a decrease in atherosclerotic plaque area in aortas from PDCD4−/−ApoE−/− mice as compared with ApoE−/− mice, by partial influence of the function of immune cells (macrophage and T-cell responses) (unpublished data). However, the role of PDCD4 in other cells that function during the formation and progression of atherosclerotic plaques remained unknown. Here, we discovered that PDCD4 protein level is low in vascular endothelial cells under physiological pulsatile shear stress both *in vivo* and *in vitro* as compared with cells under atheroprone shear stress. PDCD4 downregulation may be important in protecting aortas against atherosclerosis. PDCD4 responds to chemical stimulus as well as mechanical stress, in accordance with our previous findings that stretching human aortic smooth muscle cells *in vitro* altered PDCD4 protein expression [27].

Pulsatile shear stress was associated with downregulated PDCD4 protein level but not mRNA level, which indicates that this negative regulation is mainly post-transciptional.

In most studies of PDCD4 regulation, miR-21 is confirmed as a direct negative regulator. A bioinformatics search revealed a conserved target site for miR-21 within the PDCD4 3′ untranslated region at nucleotides 228–249 [28]. The controversial relationship between PDCD4 and miR-21 was observed in several kinds of tumor cells [20], during cardiac valvulogenesis in zebrafish [26], [29] and in stretched human aortic smooth muscle cells [27]. However, here we found that neither overexpression of miR-21 by transfecting cells with miR-21 mimics nor downregulation of miR-21 with miR-21 inhibitor altered PDCD4 protein expression in HUVECs, which agrees with Weber et al., who transfected HUVECs with pre-miR-21 but observed no significant change in PDCD4 expression [30]. In addition, pulsatile shear stress was previously found to downregulate miR-21 in HUVECs [31], which suggests a decoupling of PDCD4 and miR-21 in vascular endothelial cells and that another mechanism dominates the regulation of PDCD4 in HUVECs.

A study of human T98G cells showed that the presence of serum rapidly phosphorylated PDCD4 on Ser67 by the protein kinase p70-S6K and subsequently degraded it via the ubiquitin ligase SCF^β-TrCP^ [18], which was confirmed in HEK293 cells exposed to the tumor promoter 12-O-tetradecanoylphorbol-13-acetate [19]. In this study, PDCD4 protein level was completely rescued under pulsatile shear stress with use of the proteasome inhibitors MG-132 and lactacystin, which suggests that degradation of PDCD4 protein via proteasomes might be the most important path through which pulsatile shear stress negatively regulates PDCD4. Moreover, ubiquitination of PDCD4 was significantly increased with pulsatile shear stress, degradation of PDCD4 was blocked by silencing of β-TrCP, which further confirms the involvement of the ubiquitin-proteasome pathway. Furthermore, we observed increased phosphorylation of both Akt and p70-S6K, which agrees with previous findings that shear stress activates p70-S6K [21], [32], [33]. Subsequently, blocking the activation of Akt abolished the phosphorylation of p70-S6K, phosphorylation and degradation of PDCD4 with pulsatile shear stress. Altogether, pulsatile shear stress may activate p70-S6K via the PI3K/Akt pathway to phosphorylate PDCD4 for ubiquitin-proteasome–mediated degradation in HUVECs.

As a tumor suppressor, PDCD4 inhibits the oncogenesis and progression of cancers and improves the sensitivity of tumor cells to chemotherapy [1]. In the cardiovascular system, PDCD4 was found mainly as an inducer of apoptosis and a suppressor of proliferation [9]–[12]. Here in HUVECs, PDCD4 is shown to be an inducer of both proliferation and apoptosis. We also found that PDCD4 negatively regulated the cyclin-dependent kinase inhibitor p21^Waf1/Cip1^ in HUVECs, which agrees with the finding that siRNA knockdown of PDCD4 expression led to increased expression of p21^Waf1/Cip1^ in HCT116 colon adenocarcinoma cells [34] and human KT1 cells [35]. PDCD4 can compete with eukaryotic translation initiation factor (eIF)4G and RNA for eIF4A binding to inhibit translation initiation [3]–[6]. Whether the negative regulation of p21^Waf1/Cip1^ by PDCD4 in HUVECs is via this way warrants further investigation. The regulatory role of PDCD4 differed by cell type. In Bon-1 pancreatic neuroendocrine cells [23], ovarian cancer cells [36] and intraductal papillary mucinous neoplasm samples [37], PDCD4 acted opposite to our finding in regulating p21^Waf1/Cip1^, and in NB4 human acute promyelocytic cells, PDCD4 had no significant effect on p21^Waf1/Cip1^ expression [38].

Evidence of the role of PDCD4 in apoptosis is controversial. Although PDCD4 is considered an apoptosis inducer, siRNA knockdown of PDCD4 induced apoptosis by promoting the translation of procaspase-3 mRNA in several cancerous and non-cancerous cell lines [24]. In our study of HUVECs, PDCD4 induced apoptosis and positively regulated the activated form of caspase-3, without changing the level of caspase-3 protein itself, which indicates a pro-apoptotic role of PDCD4 by activating caspase-3.

Pulsatile shear stress helps maintain the homeostasis of vascular endothelial cells by reducing apoptosis, cell proliferation, oxidative stress and inflammation [16], [17]. Overexpression of PDCD4 by transfection with *pEGFP-C1-PDCD4* blocked the induction of p21^Waf1/Cip1^ and partly rescued proliferation with pulsatile shear stress, but did not affect the suppression of cleaved caspase-3 or reduction of apoptosis, so PDCD4 may be a positive regulator of proliferation instead of an apoptosis promoter in response to pulsatile shear stress in HUVECs.

We also observed an increase in phosphorylation of eNOS with overexpressed PDCD4 and a decrease with downregulated PDCD4, which disagrees with the finding that PDCD4 blocks the phosphorylation of Akt, whereas activated Akt phosphorylates eNOS [39], [40]. The involved mechanism warrants further investigation. Both PDCD4 overexpression and pulsatile shear stress induce phosphorylation of eNOS. Overexpression of PDCD4 with pulsatile shear stress doubled the phosphorylation of eNOS. Therefore, other mechanisms instead of PDCD4 dominate the eNOS phosphorylation induction with pulsatile shear stress.

Several studies have provided insight into the activity of the ubiquitin-proteasome pathway in response to shear stress. A proteomic study of bovine aortic endothelial cells under laminar shear stress of 15 dynes/cm^2^ showed that among various proteins, the E1-like ubiquitin-activating enzyme was greatly upregulated, whereas the ubiquitin carboxyl-terminal hydrolase was downregulated [41]. Laminar shear stress increased the phosphorylation of Mdm2, an E3 ubiquitin ligase, in rat microvascular endothelial cells [42]. The ubiquitin-proteasome pathway mediated the degradation of keratin intermediate filaments with laminar shear stress in human alveolar epithelial (A549) cells [43]. Here we addressed PDCD4 as another substrate of the ubiquitin-proteasome pathway activated by shear stress, involved in regulating turnover of endothelial cells, with the E3 ligase β-TrCP involved. However, E2s associated with shear-stress-induced PDCD4 degradation remain to be identified and the roles of the shear-induced ubiquitin-proteasome pathway in endothelial cells warrant further investigation.

In summary, the present study demonstrated that pulsatile shear stress induces the ubiquitin-proteasome–mediated degradation of PDCD4 via the PI3K/Akt pathway. PDCD4 induces turnover (proliferation and apoptosis) of HUVECs. Low PDCD4 level is associated with reduced proliferation for maintenance of HUVEC homeostasis under pulsatile shear stress.
